# Delayed-Choice Phenomena in the Projection Evolution Model

**DOI:** 10.3390/e28060640

**Published:** 2026-06-06

**Authors:** Marek Góźdź, Andrzej Góźdź, Krzysztof Lider

**Affiliations:** 1 Institute of Computer Science and Mathematics, Maria Curie-Skłodowska University, ul. Akademicka 9, 20-033 Lublin, Poland; mgozdz@kft.umcs.lublin.pl; 2 Institute of Physics, Maria Curie-Skłodowska University, pl. Marii Curie-Skłodowskiej 1, 20-031 Lublin, Poland; lider.krzysztoff@gmail.com

**Keywords:** foundations of quantum mechanics, quantum time, projection evolution model, 03.65.-w, 03.65.Ca, 03.65.Ta

## Abstract

In the Schrödinger evolution of a quantum state, time enters as a real parameter representing the coordinate. In a more consistent approach, time should be defined as a quantum observable, with the evolution taking place in a four-dimensional spacetime. This is possible in the projection evolution model in which the wave function is defined in both space and time. This allows to construct the time operator and discuss the temporal structure of the quantum processes. In this paper, we discuss a photon travelling through a Mach–Zehnder interferometer, focusing the description on the temporal profile of the wave function. We show that in this approach the delayed-choice experiments can be explained by the temporal overlap of the photon and the devices in the interferometer.

## 1. Introduction

In the standard approach to non-relativistic quantum mechanics, the time evolution of a quantum state ψ is given by the equation

(1) ψ(t,x)=U(t)ψ(x,0),

where U(t) is a unitary evolution operator generated by a Hamiltonian *H* and *t* denotes time. In the case of time-independent Hamiltonian U(t)=exp(−iHt), where here and in the following we set ℏ=1. Despite being useful in many special cases, time in this formulation is a classical parameter rather than a quantum observable. It follows that the wave function ψ is “quantized” in space but not in time, which poses interpretational problems with the spacetime formulation of the quantum theory. On the other hand, the Pauli theorem forbids to represent time as a self-adjoint operator in the space $L^{2}\left(R^{3},d^{3}x\right)$, so that Equation (1) cannot be corrected by simply replacing *t* with its operator form. Experimental work strongly suggests the need of treating time as a quantum observable. In [1], single photons were sent through a rotating disc with slits, producing an interference pattern on the screen, like in a temporal version of the double-slit experiment. This has been later confirmed in [2] in the process of photoionization at the attosecond scale, and recently in [3] in an optical setup. From the delayed-choice experiments, [4], see also the review [5], the possibility of “retro-causality” emerged; in that case, the correct description had to account for the spatial width of the particle’s wave function, making the interaction with the experimental setup possible. All quantum systems and processes, including but not limited to [6,7], are space- and time-dependent, thus a proper inclusion of time in the model is crucial.

Many attempts have been made to reformulate quantum mechanics and properly describe time in the model, including the works by Aharonov and Bohm [8], Rosenbaum [9], Grot, Rovelli, and Tate [10], Olkhovsky [11], Kijowski [12] and many others. In these papers, the authors managed to recreate the Schrödinger evolution (1) usually by postulating different interpretations of the parameter *t* and its connection to the actual time. Even though Galapon [13] showed that for a discrete spectrum Hamiltonian a self-adjoint time operator can be constructed, its consistent definition was still missing.

In this work, we will use the projection evolution approach, described in detail in [14]. In this model, time $t=x^{0}$ is the component of the four-position $x=(x^{0},\vec{x})$ and is properly represented in the form of a self-adjoint operator with eigenvalues being the time coordinates of the system. The time evolution of a quantum state is then given by a series of mappings from the current state onto the space of possible subsequent states. This generic model allows to describe unitary and non-unitary evolution scenarios as well as cases in which the Hamiltonian-based description is problematic. We focus our discussion on the delayed-choice scenario and show that the temporal part of the wave function can be used to describe photon behaviour in such setup in a natural way.

In Section 2, we describe the basic idea behind the projection evolution. We do not elaborate on all the details here, so for an extensive background and more technical discussion see [14] and references therein. In Section 3, we apply the projection evolution to a photon travelling through a Mach–Zehnder interferometer, in which the beamsplitters are inserted and removed at certain instances of time. We calculate in Section 4 the detection probabilities for both detectors, taking into account the temporal width of the photon and its temporal overlap with the beamsplitters.

## 2. The Projection Evolution

We describe the time evolution of a quantum state using the projection evolution (PEv) formalism [14]. We start from a four-dimensional formulation in which time is a quantum observable similar to the three spatial coordinates. It follows that the wave function ψ=ψ(x) depends on all four coordinates $x=(x^{0},x^{1},x^{2},x^{3})$ and the spacetime position operator ${\hat{x}}^{\mu }$ acts as a multiplication operator, i.e., ${\hat{x}}^{\mu }\psi \left(x\right)=x^{\mu }\psi \left(x\right)$. Such approach allows to describe in a consistent way different equations of motion, like the Schrödinger equation, Dirac equation, Klein–Gordon equation, and others.

The four-dimensional wave function gives the probability of finding the object at some given spacetime location, i.e., it has certain width in the spatial and in the temporal direction. This allows to define the uncertainties $\Delta \vec{x}$ and Δt, localize the object in space and time intervals, and discuss the time structure of quantum processes.

The PEv method is rooted in the earlier works by Choi [15] and Krauss [16] about quantum operations. Since for a consistent model, time has to be a quantum observable, it cannot serve as a parameter of the evolution any longer. We therefore introduce an index τ which labels subsequent steps of the quantum evolution in spacetime, observing that at each evolution step the quantum state is dependent on all four components of the position vector. The most general evolution of a quantum state is given by an operator

(2) $$E\;|:K_{\tau }\to K_{\tau +1}$$

which maps the initial Hilbert space $K_{\tau }$ at the evolution step τ onto the Hilbert space $K_{\tau +1}$ at the next step of the evolution. For the process described in this paper, the Hilbert space remains the same during the whole evolution, i.e., $K:=K_{\tau }=K_{\tau +1}$.

One needs to stress that τ is a parameter and has no direct connection with time *t*. Enumeration of events in a quantum system does not need to be in accordance with classical causality, as the wave function ψ(x) is dependent on all four spacetime coordinates *at every evolution step *τ. There is also no contradiction with the Pauli theorem, which is valid for unitary evolution operators parameterized by time only. The parameter τ is not an observable and has no physical meaning. On the other hand, having the time operator $\hat{t}$, one may build a quantum system which localizes itself on the time axis, acting effectively as a clock [17].

The evolution operators E| can have different forms, leading to different evolution equations. If E| are unitary operators we end up with the Schrödinger-like evolution. If E| are projection operators, the space K consists of all possible states on which the projections are made according to the appropriate probability distribution. In every case, the quantum state at the step τ+1 is given by the normalized action of the evolution operators $E\;{|}_{\tau +1}$ at this step onto the previous quantum state:

(3) $$\psi \left(\tau +1;t,x\right)=\frac{E\;{|}_{\tau +1}\psi \left(\tau ;t,x\right)}{∥\;E\;{|}_{\tau +1}\psi \left(\tau ;t,x\right)\;∥}.$$

The details of the PEv formalism can be found in [14,17]. The evolution operators are constructed to satisfy the Born rule. The expression (3) provides a set of all allowed states which can be reached according to the probability distribution from the previous state. All other alternatives are orthogonal to the set of the allowed states. The evolution operators are the quantum operations as introduced in [15,16].

An evolution process of a quantum state has to be described by modifications of the state ψ at different steps τ. A temporal localization of the system may happen during the evolution, in which case the state is projected onto the time axis and the time coordinate takes a definite value. In the case of no time projection, the time coordinate is smeared over a certain interval, depending on the width of the time component of the wave function. It is therefore important to remember that τ is not time and that a definite temporal coordinate can be obtained by projecting the state onto the time axis.

In our approach, the wave function describes the position of the particle in time and in space alike, i.e., at spacetime. The spatial uncertainty $\Delta \vec{x}$ comes in pair with the momentum uncertainty $\Delta \vec{p}$. The temporal uncertainty $\Delta x^{0}=\Delta t$ pairs with the temporal momentum uncertainty $\Delta p_{0}$, where $p_{0}=i\frac{\partial }{\partial t}$. Here, the $p_{0}$ operator gets the meaning of the momentum along the temporal axis and its sign represents the arrow of time, i.e., the direction of the evolution of the state. The localization of the particle in space and time comes as the result of interactions acting similarly to measurements.

## 3. The Mach–Zehnder Interferometer

**Figure 1:**
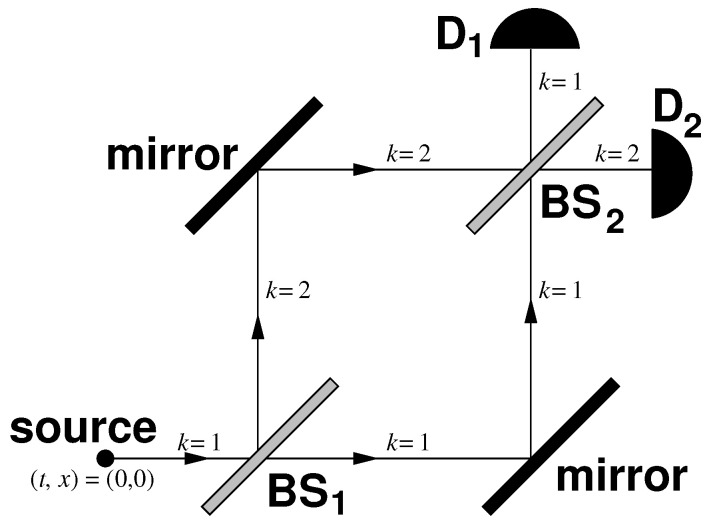
The Mach–Zehnder interferometer.

The Mach–Zehnder interferometer consists of two beamsplitters, two mirrors, and two detectors, as shown in Figure 1.

The state space of the interferometer consists of two quantum channels based on functions dependent on spacetime coordinates. Assuming channel separation, the simplified description contains two orthogonal channels in a two-dimensional spacetime, as indicated in Figure 1. In this case, we can reduce the full state space to a simpler one $K=L^{2}\left(R^{2},dt\;dx\right)⊗C^{2}$. It implies that every state $\bar{\Psi }\in K$ can be written as

(4) $$\bar{\Psi }\left(t,x\right):=\langle t,x|\bar{\Psi }\rangle =\psi \left(t,x\right)⊗\sum_{k=1}^{2}c_{k}|k\rangle ,$$

where k=1,2 labels the interferometer channels, $c_{k}\in C$, $|c_{1}{|}^{2}+{\left|c_{2}\right|}^{2}=1$, and the scalar product of two states ${\bar{\Psi }}^{\prime }$ and $\bar{\Psi }$ is given by

(5) $$\langle {\bar{\Psi }}^{\prime }|\bar{\Psi }\rangle =\int_{R^{2}}dtdx\;\psi^{\prime }{\left(t,x\right)}^{★}\psi \left(t,x\right)\sum_{k=1}^{2}{c_{k}^{\prime }}^{★}c_{k}.$$

This approach does not change the qualitative behaviour of our system.

The approximate quantized ”photon” function can be constructed by introducing electromagnetic vector fields E and H in agreement with the Maxwell equations [18]. Using the multipole expansion of the electric field,

(6) $$E\left(\vec{r}\right)=\frac{ic}{\omega }\mathrm{rot}H\left(\vec{r}\right)=\frac{ic}{\omega }\mathrm{rot}\left[f_{j}\left(r\right)Y_{m}^{j(j1)}\left(\theta ,\phi \right)\right],$$

where the spatial part takes the form of a spherical Bessel function $\frac{1}{r}\sqrt{\frac{\omega r}{c}}J_{j+1/2}\left(\frac{\omega }{c}r\right)$ and the temporal part (up to normalization) becomes $e^{i\omega_{t}t}$. The standard vector spherical harmonics describing the photon spin coupling to the angular momentum *j* are denoted by $Y_{m}^{j(j1)}\left(\theta ,\phi \right)$.

Following the full solutions for the quantized electromagnetic field [18], we use for simplification the “one-dimensional photon” (shortly photon) wave function 〈t,x|τ;γ〉=γ(τ;t,x) at the evolution step τ in a separable form, $\gamma \left(\tau ;t,x\right)=\gamma^{t}\left(\tau ;t\right)\gamma^{x}\left(\tau ;x\right)$, where the temporal part of the photon is given by the Fourier transform of an appropriate frequency profile $a(\omega_{t})$

(7) $$|\tau ;\gamma^{t}\rangle \to \gamma^{t}\left(\tau ;t\right)=\frac{1}{\sqrt{2\pi }}\int_{R}d\omega_{t}a\left(\omega_{t}\right)e^{i\omega_{t}\left(t-\alpha \left(\tau \right)\right)},$$

with $\omega_{t}$ describing the temporal width of the photon’s profile. This form leads to a uniform probability distribution in time. In the lowest order of the Bessel function, the spatial part can be written as

(8) $$|\tau ;\gamma^{x}\rangle \to \gamma^{x}\left(\tau ;x\right)=\sqrt{\frac{\omega_{x}}{\pi }}\frac{\sin(\omega_{x}\left(x-\alpha \left(\tau \right)\right))}{\omega_{x}\left(x-\alpha \left(\tau \right)\right)},$$

where the parameter $\omega_{x}$ represents the spatial width of the photon’s profile. Using this or any other form of the phenomenological photon wave function does not change qualitatively our discussion of the delayed-choice experiment.

To avoid unnecessary complication of the description, we consider a semiclassical motion of the photon as a shift in spacetime. Following this approach, the maximum of the state (8) shifts during the evolution along the *x* axis, simulating the one-dimensional semiclassical motion of the photon. These shifts are given by cα(τ), which for setting the speed of light c=1 simplifies to α(τ) and corresponds to the motion along the world line x=t. This also represents the spatial and temporal localization of the maxima of the photon wave function at the evolution step τ.

In the following, we write only these evolution operators which describe our process and we omit all other alternatives. The evolution of the state of the photon can be described by a sequence of interactions with the elements of the interferometer and propagation between them. We may list the following seven steps: at $\tau =\tau_{0}$ we start with the input state $|\bar{\Psi }\rangle_{in}$; at $\tau =\tau_{1}$ the photon reaches the first entrance channel of the interferometer; at $\tau =\tau_{2}$ the photon may interact with the first beamsplitter BS_1_; at $\tau =\tau_{3}$ the photon reaches the mirrors and at $\tau =\tau_{4}$ interacts with them; at $\tau =\tau_{5}$ it is shifted to the second beamsplitter BS_2_, and at $\tau =\tau_{6}$ it may interact with it; and at $\tau =\tau_{7}$ the photon arrives to the detectors and is measured.

The photon is produced in the source at $\tau_{0}$ in the first channel, so the first evolution operator reads

(9) $$E\;|\left(\tau_{0};1\right)=|\tau_{0};\gamma \rangle \langle \tau_{0};\gamma |⊗|1\rangle \langle 1|.$$

Acting on the input state $|\bar{\Psi }\rangle_{in}$ this operator determines the initial state of the photon as

(10) $$|\bar{\Psi }\left(\tau_{0}\right)\rangle =|\tau_{0};\gamma \rangle ⊗|1\rangle$$

Here, we assume the initial shift $\alpha (\tau_{0})=0$.

As it was mentioned above, the free motion of the photon is described by a simple shift in the spacetime along the world line x=t. This implies the following evolution operator:

(11) $$E\;|\left(\tau ;F\right)=\hat{T}\left(\alpha \left(\tau \right)\right)⊗\hat{1\;1},$$

where $\hat{T}\left(\alpha \right)$ is the spacetime translation operator in K. This means that

(12) $$\hat{T}\left(\alpha \right)\bar{\Psi }\left(t,x\right)=\bar{\Psi }\left(t-\alpha ,x-\alpha \right).$$

Reaching the beamsplitter BS_1_ the photon state is

(13) $$|\bar{\Psi }\left(\tau_{1}\right)\rangle =E\;|\left(\tau_{1};F\right)|\tau_{0};\gamma \rangle ⊗|1\rangle =|\tau_{1};\gamma \rangle ⊗|1\rangle .$$

In general, the beamsplitter could modify the spacetime profile of the photon. As this plays no role in our analysis, we will not take it into account. In constructing the evolution operator we have to take into account that the beamsplitter will influence the photon only if there is a non-zero spacetime overlap between their wave functions. It means that if the photon coexists with the beamsplitter in spacetime, its wave function will be modified even if the beamsplitter will earlier or later be removed from the system. The details depend here on the profiles of the temporal parts of the photon and beamsplitter wave functions.

For further purpose, we introduce the Boolean function χ(C) defined as follows: χ(C)=1 if the condition C is fulfilled, otherwise this function is equal to zero.

Using this function, we propose the appropriate evolution operator in the form

(14) $$E\;|\left(\tau ;{\mathrm{BS}}_{l}\right):=P\left(\Omega_{l}\right)⊗{\hat{B}}_{l}+\left(1-P\left(\Omega_{l}\right)\right)⊗\hat{1\;1},$$

where the Lebesgue–Stieltjes integral

(15) $$P\left(\Omega \right):=\int_{R^{2}}dtdx\;|t,x\rangle \chi \left(\left(t,x\right)\in \Omega \right)\langle t,x|$$

projects onto the subset Ω of the spacetime positions. The set $\Omega_{l}$, l=1,2, describes the spacetime profile of the lth beamsplitter, i.e., it determines when and where the beamsplitter is present in the system.

The operators ${\hat{B}}_{l}$ mix channels and are represented for BS_1_ and BS_2_ in the channel basis {|1〉,|2〉} by

(16) $$\begin{array}{ccc} & & {\hat{B}}_{1}|1\rangle =\frac{1}{\sqrt{2}}(|1\rangle +|2\rangle ),\;{\hat{B}}_{1}|2\rangle =\frac{1}{\sqrt{2}}(-|1\rangle +|2\rangle ), \end{array}$$

(17) $$\begin{array}{ccc} & & {\hat{B}}_{2}|1\rangle =\frac{1}{\sqrt{2}}(|1\rangle -|2\rangle ),\;{\hat{B}}_{2}|2\rangle =\frac{1}{\sqrt{2}}(|1\rangle +|2\rangle ). \end{array}$$

After the first beamsplitter, the normalized state is given by

(18) $$\bar{\Psi }\left(\tau_{2}\right)\left(t,x\right)=\chi \left(\left(t,x\right)\in \Omega_{1}\right)\langle t,x|\tau_{1};\gamma \rangle ⊗\frac{1}{\sqrt{2}}\left(|1\rangle +|2\rangle \right)+\chi \left(\left(t,x\right)\notin \Omega_{1}\right)\langle t,x|\tau_{1};\gamma \rangle ⊗|1\rangle .$$

Again, the free evolution shifts the resulting state to the next part of the interferometer and the state entering the mirrors is given by

(19) $$\begin{array}{c} \bar{\Psi }\left(\tau_{3}\right)\left(t,x\right)=\hat{T}\left(\alpha \left(\tau_{3}\right)-\alpha \left(\tau_{1}\right)\right)\bar{\Psi }\left(\tau_{2}\right)\left(t,x\right)\\ =\chi \left(\left(t-\left(\alpha_{3}-\alpha_{1}\right),x-\left(\alpha_{3}-\alpha_{1}\right)\right)\in \Omega_{1}\right)\langle t,x|\tau_{3};\gamma \rangle ⊗\frac{1}{\sqrt{2}}\left(|1\rangle +|2\rangle \right)\\ +\chi \left(\left(t-\left(\alpha_{3}-\alpha_{1}\right),x-\left(\alpha_{3}-\alpha_{1}\right)\right)\notin \Omega_{1}\right)\langle t,x|\tau_{3};\gamma \rangle ⊗|1\rangle , \end{array}$$

where $\alpha_{k}\equiv \alpha \left(\tau_{k}\right)$.

The mirrors are large enough to prevent the photons from passing around them. Let us assume that the mirrors can modify phases of the photon in each arm of the interferometer independently. The evolution operator E|(τ;M) is diagonal in the channel basis:

(20) $$E\;|\left(\tau ;M\left(\kappa_{1},\kappa_{2}\right)\right)=\hat{1\;1}⊗\hat{M}\left(\kappa_{1},\kappa_{2}\right),$$

The operator $\hat{M}\left(\kappa_{1},\kappa_{2}\right)$ acts on the basic channel states as

(21) $$\hat{M}\left(\kappa_{1},\kappa_{2}\right)|k\rangle =e^{i\kappa_{k}}|k\rangle .$$

The mirrors change the previous state as follows:

(22) $$\begin{array}{c} \bar{\Psi }\left(\tau_{4};t,x\right)=\chi \left(\left(t-\left(\alpha_{3}-\alpha_{1}\right),x-\left(\alpha_{3}-\alpha_{1}\right)\right)\in \Omega_{1}\right)\langle t,x|\tau_{3};\gamma \rangle ⊗\frac{1}{\sqrt{2}}\left(e^{i\kappa_{1}}|1\rangle +e^{i\kappa_{2}}|2\rangle \right)\\ +\chi \left(\left(t-\left(\alpha_{3}-\alpha_{1}\right),x-\left(\alpha_{3}-\alpha_{1}\right)\right)\notin \Omega_{1}\right)\langle t,x|\tau_{3};\gamma \rangle ⊗e^{i\kappa_{1}}|1\rangle . \end{array}$$

The free motion to the beamsplitter BS_2_ prepares its input state as

(23) Ψ¯(τ5;t,x)=T^(α(τ5)−α(τ3))Ψ¯(τ4;t,x)=χ((t−(α5−α1),x−(α5−α1))∈Ω1)〈t,x|τ5;γ〉⊗12eiκ1|1〉+eiκ2|2〉+χ((t−(α5−α1),x−(α5−α1))∉Ω1)〈t,x|τ5;γ〉⊗eiκ1|1〉xx.

At the evolution step $\tau_{6}$, the beamsplitter BS_2_ mixes channels which results in a possible interference:

(24) $$\begin{array}{c} \bar{\Psi }\left(\tau_{6};t,x\right)=\chi \left(\left(t-\left(\alpha_{5}-\alpha_{1}\right),x-\left(\alpha_{5}-\alpha_{1}\right)\right)\in \Omega_{1}\right)\chi \left(\left(t,x\right)\in \Omega_{2}\right)\langle t,x|\tau_{5};\gamma \rangle \\ ⊗\frac{1}{2}\{\left(e^{i\kappa_{1}}+e^{i\kappa_{2}}\right)|1\rangle +\left(-e^{i\kappa_{1}}+e^{i\kappa_{2}}\right)\left|2\rangle \right\}\\ +\chi \left(\left(t-\left(\alpha_{5}-\alpha_{1}\right),x-\left(\alpha_{5}-\alpha_{1}\right)\right)\notin \Omega_{1}\right)\chi \left(\left(t,x\right)\in \Omega_{2}\right)\langle t,x|\tau_{5};\gamma \rangle ⊗\frac{e^{i\kappa_{1}}}{\sqrt{2}}(|1\rangle -\left|2\rangle \right)\\ +\chi \left(\left(t-\left(\alpha_{5}-\alpha_{1}\right),x-\left(\alpha_{5}-\alpha_{1}\right)\right)\in \Omega_{1}\right)\chi \left(\left(t,x\right)\notin \Omega_{2}\right)\langle t,x|\tau_{5};\gamma \rangle ⊗\frac{1}{\sqrt{2}}(e^{i\kappa_{1}}|1\rangle +e^{i\kappa_{2}}\left|2\rangle \right)\\ +\chi \left(\left(t-\left(\alpha_{5}-\alpha_{1}\right),x-\left(\alpha_{5}-\alpha_{1}\right)\right)\notin \Omega_{1}\right)\chi \left(\left(t,x\right)\notin \Omega_{2}\right)\langle t,x|\tau_{5};\gamma \rangle ⊗e^{i\kappa_{1}}|1\rangle . \end{array}$$

The state (24) describes four independent basic scenarios. For all of them the next evolution step is a free motion of the photon to the detectors. It is described by the vector $|\bar{\Psi }\left(\tau_{7}\right)\rangle =\hat{T}\left(\alpha \left(\tau_{7}\right)-\alpha \left(\tau_{5}\right)\right)|\bar{\Psi }\left(\tau_{6}\right)\rangle$ which has the same structure as (24). The vector $|\bar{\Psi }\left(\tau_{7}\right)\rangle$ is the final state of the photon before its measurement by the detectors. The most important information is the probability distribution of detecting the photon in the detector $D_{k}$ which monitors the channel |k〉, k=1,2.

In our case, each detector is represented the projection operator $P(D_{k})$ which localizes the photon in the time interval $\left(\bar{t}-\epsilon_{t}/2,\bar{t}+\epsilon_{t}/2\right)$ and at the spatial position in the interval $\left(\bar{x}-\epsilon_{x}/2,\bar{x}+\epsilon_{x}/2\right)$. Similarly to the mirrors we assume that the photon cannot pass the detectors undetected. The evolution operators read

(25) $$P\left(D_{k}\right)=\int_{\bar{t}-\epsilon_{t}/2}^{\bar{t}+\epsilon_{t}/2}dt\;\int_{\bar{x}-\epsilon_{x}/2}^{\bar{x}+\epsilon_{x}/2}dx\;|t,x\rangle ⊗\left|k\rangle \langle k\right|⊗\langle t,x|,$$

where |t,x〉 are eigenstates of the time and position operators in $L^{2}\left(R^{2},dt\;dx\right)$ represented by the standard multiplication type operators: $\hat{t}f\left(t,x\right)=tf\left(t,x\right)$ and $\hat{x}f\left(t,x\right)=xf\left(t,x\right)$ [14].

Notice that the operators (25) are an orthogonal resolution of unity. $\sum P(D_{k})$ over all spacetime regions and both channels is equal to the unit operator in our state space. This feature normalizes calculated probabilities to 1, as required.

The required probability of finding the photon in the detector *k* at time $t\in (\bar{t}-\epsilon_{t}/2,\bar{t}+\epsilon_{t}/2)$ is given by the standard formula

(26) $$\mathrm{Prob}\;\left(D_{k};\bar{t},\bar{x}\right)=\langle \bar{\Psi }\left(\tau_{7}\right)|P\left(D_{k}\right)|\bar{\Psi }\left(\tau_{7}\right)\rangle =\int_{\bar{t}-\epsilon_{t}/2}^{\bar{t}+\epsilon_{t}/2}dt\;\int_{\bar{x}-\epsilon_{x}/2}^{\bar{x}+\epsilon_{x}/2}dx\;|(\langle t,x|⊗\langle k|)|\bar{\Psi }\left(\tau_{7}\right){\rangle |}^{2}.$$

Using appropriately shifted states (24), the spacetime representation of the measured state is

(27) $$\begin{array}{c} \left(\langle t,x|⊗\langle k|)\right|\bar{\Psi }\left(\tau_{7}\right)\rangle =\chi_{71}\chi_{75}\gamma \left(\tau_{7};t,x\right)\frac{1}{2}\left\{\left(e^{i\kappa_{1}}+e^{i\kappa_{2}}\right)\delta_{k1}+\left(-e^{i\kappa_{1}}+e^{i\kappa_{2}}\right)\delta_{k2}\right\}\\ +\bar{\chi_{71}}\chi_{75}\gamma \left(\tau_{7};t,x\right)\frac{e^{i\kappa_{1}}}{\sqrt{2}}\left(\delta_{k1}-\delta_{k2}\right)+\chi_{71}\bar{\chi_{75}}\gamma \left(\tau_{7};t,x\right)\frac{1}{\sqrt{2}}\left(e^{i\kappa_{1}}\delta_{k1}+e^{i\kappa_{2}}\delta_{k2}\right)\\ +\bar{\chi_{71}}\bar{\chi_{75}}\gamma \left(\tau_{7};t,x\right)e^{i\kappa_{1}}\delta_{k1}, \end{array}$$

where we have denoted

$$\begin{array}{c} \chi_{71}\equiv \chi \left(\left(t-\left(\alpha_{7}-\alpha_{1}\right),x-\left(\alpha_{7}-\alpha_{1}\right)\right)\in \Omega_{1}\right),\\ \bar{\chi_{71}}\equiv \chi \left(\left(t-\left(\alpha_{7}-\alpha_{1}\right),x-\left(\alpha_{7}-\alpha_{1}\right)\right)\notin \Omega_{1}\right),\\ \chi_{75}\equiv \chi \left(\left(t-\left(\alpha_{7}-\alpha_{5}\right),x-\left(\alpha_{7}-\alpha_{5}\right)\right)\in \Omega_{2}\right).\\ \bar{\chi_{75}}\equiv \chi \left(\left(t-\left(\alpha_{7}-\alpha_{5}\right),x-\left(\alpha_{7}-\alpha_{5}\right)\right)\notin \Omega_{2}\right). \end{array}$$

Again, we get four independent alternatives instead of sixteen combinations of matrix elements. This is due to the fact that the Boolean function χ(C) is a projector, i.e., $\chi {\left(\mathrm{true}\right)}^{2}=\chi \left(\mathrm{true}\right)=1$ and χ(true)χ(false)=0. The density probability function in (26) can be now written as

(28) $$\begin{array}{c} \left|(\langle t,x|⊗\langle k|)\right|\bar{\Psi }\left(\tau_{7}\right){\rangle |}^{2}=|\gamma \left(\tau_{7};t,x\right){|}^{2}\{\chi_{71}\chi_{75}\frac{1}{2}[\left(1+\cos\left(\kappa_{1}-\kappa_{2}\right)\right)\delta_{k1}\\ +\left(1-\cos\left(\kappa_{1}-\kappa_{2}\right)\right)\delta_{k2}]+\frac{1}{2}\bar{\chi_{71}}\chi_{75}\left(\delta_{k1}+\delta_{k2}\right)+\frac{1}{2}\chi_{71}\bar{\chi_{75}}\left(\delta_{k1}+\delta_{k2}\right)+\bar{\chi_{71}}\bar{\chi_{75}}\delta_{k1}\}. \end{array}$$

One needs to remember that $\alpha_{k}=\alpha \left(\tau_{k}\right)$ represents the distance from the source of the photon to the appropriate point in the interferometer.

## 4. Discussion

We assume that the photon enters the interferometer at (t,x)=(0,0), and that the first beamsplitter, the mirrors, the second beamsplitter, and the detectors are all five units of space apart, i.e., BS_1_ can be reached at x=5, the mirrors at x=10, BS_2_ at x=15, and the detectors at x=20. In the following we assume for the mirrors $\kappa_{1}=\kappa_{2}=\pi$.

If there is only one beamsplitter present in the system, the wave function of the photon is statistically split into both channels, reaching both of the detectors with probability $\frac{1}{2}$. If there is no beamsplitter present, only the detector D_1_ will detect the photon. If both beamsplitters interact with the photon, the interference will enhance the signal in D_1_ and destroy the signal in D_2_.

If the beamsplitters are removed and inserted in the setup, the photon may or may not react to the change. This is called the delayed-choice experiment and it is usually explained invoking the spatial width of the wave function. If the photon has a non-zero overlap with the beamsplitter, it will modify its state in accordance with the changes made to the setup. In our approach, however, time enters the photon’s position four-vector and should also be considered. Below we discuss some delayed-choice scenarios focusing on the temporal interaction and show that the results are fully compatible with the expectations.

### 4.1. The Symmetric Case

The behaviour of the photon will depend on its spatial and temporal profiles. Let us start with the photon wave function in the shape of a box, with sharp boundaries, both in space and time. The normalized step function centred around $x_{0}$ is given by

(29) $$\mathrm{step}\left(x-x_{0}\right)=\left\{\begin{array}{cc} \frac{1}{\sqrt{\Delta_{x}}}, & x_{0}-\frac{\Delta_{x}}{2}<x<x_{0}+\frac{\Delta_{x}}{2}\\ 0, & \mathrm{otherwise} \end{array}\right.$$

and similarly for time

(30) $$\mathrm{step}\left(t-t_{0}\right)=\left\{\begin{array}{cc} \frac{1}{\sqrt{\Delta_{t}}}, & t_{0}-\frac{\Delta_{t}}{2}<t<t_{0}+\frac{\Delta_{t}}{2}\\ 0, & \mathrm{otherwise} \end{array}\right..$$

The photon will interact with everything from within the boxes, so if the beamsplitter appears earlier or later than the position $\left(t_{0},x_{0}\right)$ but has a non-zero overlap with the photon’s profile, it will affect the particle. The rectangular box, even though artificial, serves as a good illustration of the mechanism of the photon interaction with the experimental setup. Due to the existence of sharp boundaries, the spacetime region occupied by the particle is always clearly defined.

In a more physically viable case, the temporal rectangular box can be replaced by a normalized Gaussian

(31) $$|\gamma^{t}{\left(t\right)|}^{2}=\frac{1}{\sqrt{2\pi }\omega_{t}}e^{-\frac{t^{2}}{2\omega_{t}^{2}}}$$

and the spatial part by the lowest order of Equation (8). This photon is symmetric both in the forward and in the backward direction of time. It means that the changes made to the system before and after the photon has reached the required spacetime point can still alter the quantum state. This remark is valid for all the elements in the interferometer: the beamsplitters, the mirrors, and even the detectors. The projection evolution model does not distinguish between them.

**Figure 2:**
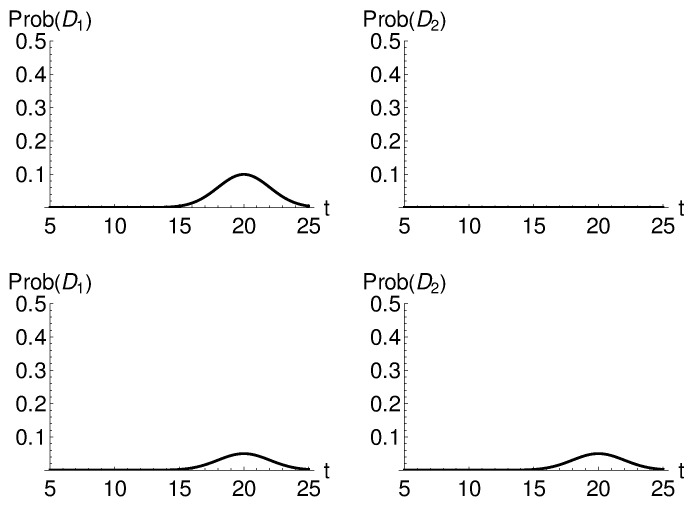
The symmetric photon time profile. The detection distribution probabilities for none or both beamsplitters present in the setup (**upper figure**); for only one of the beamsplitters present in the setup (**lower figure**).

In Figure 2, the detection probabilities for detectors D_1_ and D_2_ are presented. One notices that the maximum detection probability appears for $\left(t_{0},x_{0}\right)=\left(20,20\right)$, i.e., for the spacetime location of the detector. The same result will be obtained for the photon being treated as a classical point-like particle. The position of the classical particle is represented by the position of the maximal detection probability of the photon, which in the case of symmetric profile, coincides with the position of the global maximum.

### 4.2. The Asymmetric Case

The other possibility is the asymmetric case in which the photon has a strong temporal maximum with a tail directed forwards or backwards in time. It may be given in the form

(32) $$|\gamma^{t}{\left(t\right)|}^{2}=\omega_{t}^{2}\chi \left(\pm t\geq 0\right)\left(\pm t\right)e^{-\omega_{t}\left(\pm t\right)}$$

and represents the situation in which the particle either senses the time interval before its maximum or can probe later times.

**Figure 3:**
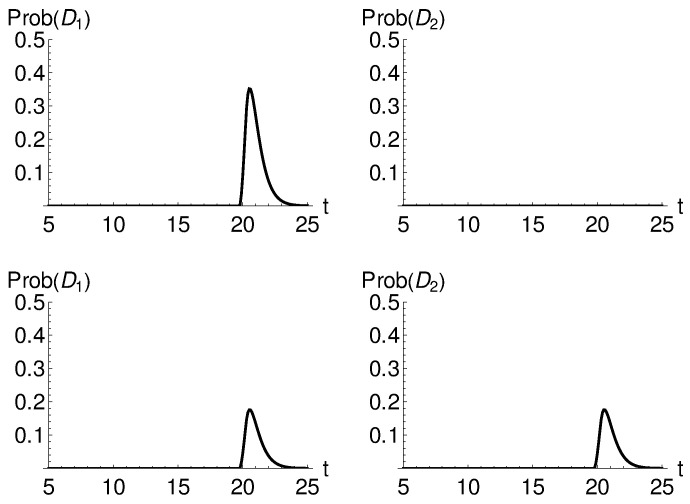
The asymmetric photon profile with a tail forward in time. The detection distribution probabilities for none or both beamsplitters present in the setup (**upper figure**); for only one of the beamsplitters present in the setup (**lower figure**).

**Figure 4:**
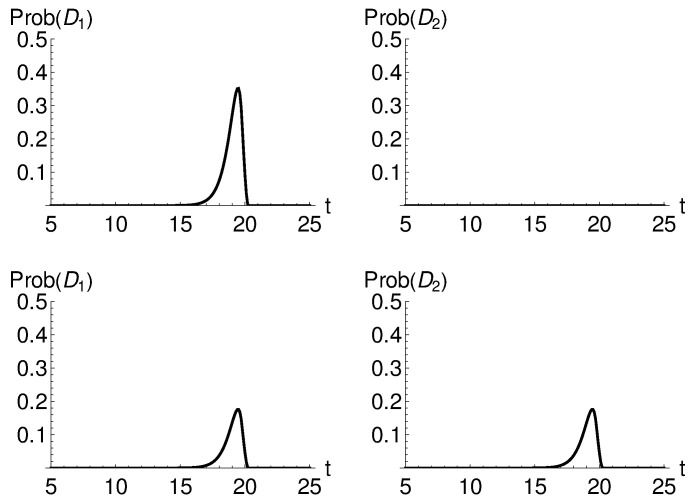
The asymmetric photon profile with a tail backwards in time. The detection distribution probabilities for none or both beamsplitters present in the setup (**upper figure**); for only one of the beamsplitters present in the setup (**lower figure**).

In these cases, the changes made to the interferometer will only affect the photon if they appear in the correct time interval. If the photon has a backwards time tail, it will react to all the changes made before it has reached the given spacetime point. It means that it will know about all the manipulations of the beamsplitters before reaching their spacetime localization. Contrary, the photon with a time tail directed forward in time will react to the changes made after it has passed the given spacetime location. This is valid not only for the experimental setup, but also for the detectors, which means that a photon with a forward time tail has a probability to be detected earlier than the photon with a backward time tail. This is illustrated in Figure 3 and Figure 4.

### 4.3. Other Examples

We will present below a few special cases in which the beamsplitters are inserted in the interferometer before or after the photon has reached its spatial location. In all of the cases, the temporal parts of the photon wave function give the possibility of interaction with the beamsplitter. This, alongside the spatial interaction, gives a valid explanation of the delayed-choice experiments.

**Figure 5:**
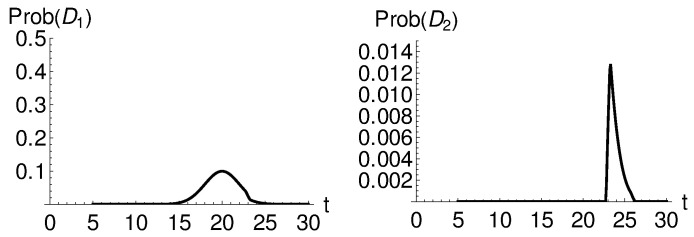
The detection distribution probabilities in Scenario 1.

*Scenario 1:* In the first scenario BS_1_ is absent and BS_2_ is present for 18≤t≤21. The temporal part of the photon is a Gaussian. In this case, a small detection probability appears in the second detector for times greater than t=20 (see Figure 5).

**Figure 6:**
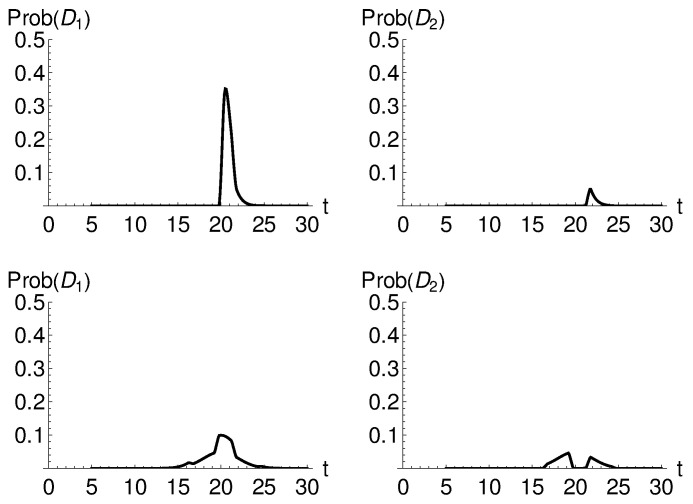
The detection distribution probabilities in Scenario 2 for a forward-directed temporal profile (**upper figure**) and a Gaussian (**lower figure**).

*Scenario 2:* The first beamsplitter is present before the photon can reach its spatial position, 1.5≤t≤4.5. The second beamsplitter appears later, 16.5≤t≤19.5. The photon has a temporal tail directed forwards. In this case, the second detector gets a small probability of detection (see Figure 6). We notice that if the photon had the temporal tail in the backwards direction, $D_{2}$ would not react as the photon could not react with BS_2_.

**Figure 7:**
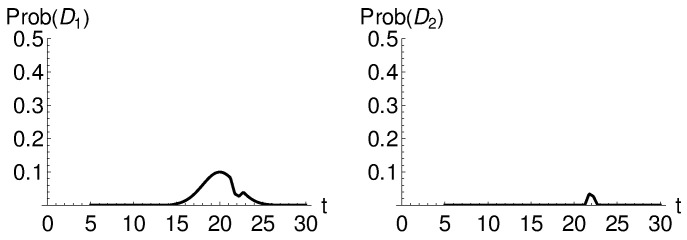
The detection distribution probabilities in Scenario 3.

*Scenario 3:* The BS_1_ is present at 6.5≤t≤7.5 and the BS_2_ at 6.5≤t≤9.5, so we have a situation when only one, then both, and again only one beamsplitter is present in the system. A Gaussian photon will have the detection probabilities as shown in Figure 7.

## 5. Closing Remarks

The free parameter $\omega_{t}$ controls the width of the temporal profile. To our best knowledge, there is no direct experimental data on this subject, but in normal conditions we expect the temporal widths to be very small. We believe that appropriately performed temporal interference experiments, like those already mentioned [1,2,3], have the potential to investigate the shape of the temporal part of the particles’ wave functions. A modification of the Aspect delayed-choice experiment [4] may be useful to study the cases described in Section 4. In any case, the temporal part of the wave function provides a natural way to describe the time structure of quantum events like the temporal interference, time-of-arrival, nuclear fission and fusion processes, time structure of elementary particle interactions and many more. In this paper, we have shown that the delayed-choice experiment can be successfully described by the temporal interaction on the example of a Mach–Zehnder interferometer.

## Data Availability

Data is contained within the article.
